# Using geovisual analytics in Google Earth to understand disease distribution: a case study of campylobacteriosis in the Czech Republic (2008–2012)

**DOI:** 10.1186/1476-072X-14-7

**Published:** 2015-01-28

**Authors:** Lukáš Marek, Pavel Tuček, Vít Pászto

**Affiliations:** Department of Geoinformatics, Faculty of Science, Palacky University in Olomouc, 17.listopadu 50, 77146 Olomouc, Czech Republic

**Keywords:** Google Earth™, Space-time pattern, Spatio-temporal interpolation, Campylobacteriosis, Czech Republic, Interactive visualisation, Clustering

## Abstract

**Background:**

Visual analytics aims to connect the processing power of information technologies and the user’s ability of logical thinking and reasoning through the complex visual interaction. Moreover, the most of the data contain the spatial component. Therefore, the need for geovisual tools and methods arises. Either one can develop own system but the dissemination of findings and its usability might be problematic or the widespread and well-known platform can be utilized. The aim of this paper is to prove the applicability of Google Earth™ software as a tool for geovisual analytics that helps to understand the spatio-temporal patterns of the disease distribution.

**Methods:**

We combined the complex joint spatio-temporal analysis with comprehensive visualisation. We analysed the spatio-temporal distribution of the campylobacteriosis in the Czech Republic between 2008 and 2012. We applied three main approaches in the study: (1) the geovisual analytics of the surveillance data that were visualised in the form of bubble chart; (2) the geovisual analytics of the disease’s weekly incidence surfaces computed by spatio-temporal kriging and (3) the spatio-temporal scan statistics that was employed in order to identify high or low rates clusters of affected municipalities. The final data are stored in Keyhole Markup Language files and visualised in Google Earth™ in order to apply geovisual analytics.

**Results:**

Using geovisual analytics we were able to display and retrieve information from complex dataset efficiently. Instead of searching for patterns in a series of static maps or using numerical statistics, we created the set of interactive visualisations in order to explore and communicate results of analyses to the wider audience. The results of the geovisual analytics identified periodical patterns in the behaviour of the disease as well as fourteen spatio-temporal clusters of increased relative risk.

**Conclusions:**

We prove that Google Earth™ software is a usable tool for the geovisual analysis of the disease distribution. Google Earth™ has many indisputable advantages (widespread, freely available, intuitive interface, space-time visualisation capabilities and animations, communication of results), nevertheless it is still needed to combine it with pre-processing tools that prepare the data into a form suitable for the geovisual analytics itself.

**Electronic supplementary material:**

The online version of this article (doi:10.1186/1476-072X-14-7) contains supplementary material, which is available to authorized users.

## Background

### Rise of visual analytics

The exploration of the spatial distribution of diseases and their patterns became the relevant research in both, medical sciences and geosciences. It can help to understand not only the spread or location of the disease, but it can also address potential environmental and/or social factors that cause the higher occurrence of the disease. The increasing amount of (geo)data and their complexity coerced into the need for complex tools and methods that enable the connection of computing power of information technologies and the human reasoning. The scientific field and theory of visual analytics is capable of fulfilling these requirements. By visual analytics, it is usually meant the science of analytical reasoning facilitated by interactive visual interfaces [1]. A more sophisticated description of this emerging scientific field describes the complexity and the dynamic nature of the area more appropriately as it combines automated analysis techniques with interactive visualisations for an effective understanding, reasoning and decision making on the basis of very large and complex datasets [2]. The goal of visual analytics is to make the processes of data elaboration, information gathering and knowledge generation transparent to tool users [3]. To meet these goals, research methods of visual analytics identify three major directions that focus on the analytical reasoning; (1) visual representation and interaction; (2) data representations and transformations; and (3) production, presentation and dissemination of results [1, 4].

### Geovisual analytics

Nowadays, most of the data also contain the spatial component, so the traditional visual analytics needs to be enhanced, and the new sub-discipline called geovisual analytics emerges. Geovisual analytics is then described as the science of analytical reasoning and decision-making with geographic information, facilitated by interactive visual interfaces, computational methods, and knowledge construction, representation and management strategies [5]. The end goal of the investigation using geovisual analytics techniques should be oriented on the dissemination of results to decision makers while providing the succinct communication of the interpretations made by analysts [6]. It is worth to notice that the time component holds at least the same importance as space within the geovisual evaluation of the phenomena.

The rising popularity of the (geo)visual analytics in the research, education and also among the general public supports the development of specialized complex software tools, either desktop or web-based. GeoViz Toolkit [7] is one of the user-friendly desktop applications that were developed by GeoVista Center of The Pennsylvania State University. GeoDa Center for geospatial analysis and computation is another provider of geovisual analytics software with linked view. One can mention mainly GeoDa; a free, open source, cross-platform software program that serves as an introduction to exploratory spatial data analysis [8]. The Organisation for Economic Co-operation and Development (OECD) and Eurostat provide visually attractive online platforms for geovisual analytics that are well supplied with mainly statistical data including some health related topics. Both platforms, OECD Regional eXplorer [9] and Eurostat Regional Statistics Illustrated [10] aim to provide the data and their visualisation to the public. Their customization and data upload are limited to the data and tools originally prepared on web pages. StatPlanet [11] is more advanced web-based interactive data visualisation and mapping application that also allows the customization for the user’s purpose as well as uploading the data. Victorian Heart Maps [12] are one of the real-world examples of StatPlanet application with the health data. One can also use well-known Gapminder [13], Pivot [14] or create user’s geovisual applications using the capabilities of ArcGIS Online platform [15].

### Google and its technologies

Google, as one of the recent technological leaders, also develops tools enabling the data browsing and charting (Google Public Dataset Directory [16]) or mapping and visual exploring (Google Fusion Tables [17]). In this paper, we demonstrate a geovisual analytics possibilities of Google Earth™ desktop application [18]. Google Earth™ is a popular virtual globe application that allows displaying of spatial data and their interactive exploring. Despite the fact that Google Earth™ is not the fully-operational platform for geovisual analytics, we still consider it capable of fulfilling the several of visual analytics primary goals – the exploration of (unknown) data patterns, the dissemination of results and the communication of their interpretations. However, one has to be aware that the interpretation of data results, as well as spatio-temporal thinking and reasoning, are complex processes that require not only the focus user’s mind, but they are also experience-dependent. The main reasons, why Google Earth™ was utilized in this study can be summarized as (1) the software is free of charge (we do not require Pro version); (2) it is well-known to public and probably the most widespread browser of geodata (more than 1 billion downloads [19]); (3) it is easy to use and considered intuitive; (4) it provides high-quality remote sensing imagery and administrative data; (5) it supports of KML (Keyhole Markup Language) file format, which is XML-based file format used to display geodata that is also the OGC (Open Geospatial Consortium) standard for the exchange of spatial data. The applicability of the platform in the geohealth research is documented by previous studies and papers [20–22]. The comprehensive comparison of Google Earth™ versus commonly used GIS software provides [23].

### Case study

The suitability of the Google Earth™ for the geovisual analytics of health datasets is shown in the case study. The case study combines the spatio-temporal analysis of the disease distribution with its geovisual exploration. It focuses on the distribution of campylobacteriosis in the Czech Republic between 2008 and 2012. Campylobacteriosis is one of the most common gastroenteritis of humans. Most of the campylobacteriosis cases are caused by Campylobacter jejuni, which is widespread in different environments but is often linked to the poultry and raw meat. Previous studies estimated that the disease is highly underreported, which may be caused by the fact that the disease can sometimes have mild symptoms. Approximately 72% of municipalities recorded at least one case of the disease during the analysed period. The occurrence of the disease, as well as its incidence, grew gradually until the year 2010 when the peak was recorded (see Table 1 for more details). The disease occurrence and incidence started to decrease since then. Using the Google Earth™ platform, we wanted to explore how the disease distribution pattern has been changing during the observed period in the Czech Republic and also in its particular regions.

**Table 1:** **Basic statistics of campylobacteriosis frequency and smoothed incidence in the Czech Republic in years 2008–2012**

	2008		2009		2010		2011		2012		Overall	
	Freq.	Inc.	Freq.	Inc.	Freq.	Inc.	Freq.	Inc.	Freq.	Inc.	Freq.	Inc.
Minimum	0.00	0.00	0.00	0.00	0.00	0.00	0.00	0.00	0.00	0.00	0.00	0.00
Maximum	480.00	7,750.46	412.00	4,049.63	403.00	3,532.49	339.00	7,472.88	363.00	7,892.20	396.00	6,605.42
Median	0.00	142.14	0.00	153.50	0.00	157.03	0.00	141.94	0.00	145.44	0.40	144.38
Mean	3.14	164.63	3.19	171.72	3.31	179.38	2.94	156.72	2.88	161.52	3.09	161.72
Std.Dev.	16.90	165.62	15.82	145.71	16.45	145.02	14.06	149.15	13.19	150.61	15.08	138.85
Sum	20,076		20,348		21,150		18,797		18,393		19,752	

The table shows selected basic statistical characteristics of the occurrence frequency (Freq.; no. of cases) and the disease’s incidence (Inc.; no. of cases per 100,000 population) in municipalities in the Czech Republic. Statistics are computed for individual years and also for all years together (Overall). The abbreviation Std. Dev. stands for the standard deviation.

**Figure 1:**
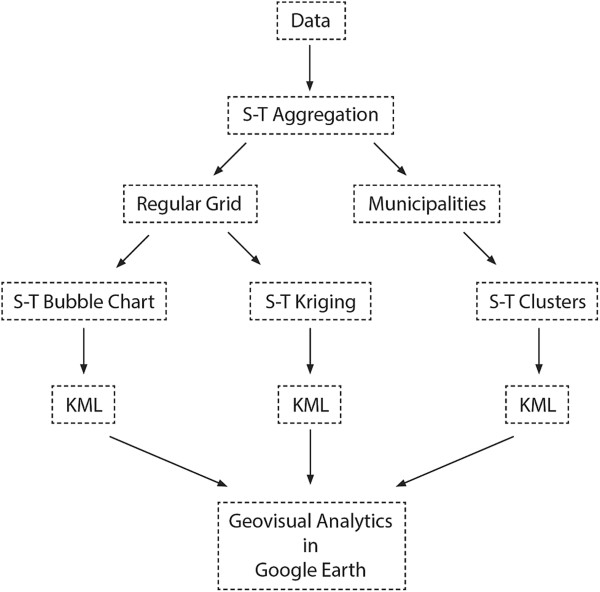
**The workflow of the case study.**

The pre-processing of the data, all analyses and the preparation of results for the visualisation proceeded in free or open source software. QGIS was utilized for the preparation of spatial data. Most of the analytical work and the generation of final KML files were made using *R programming language 3.1.0* with suitable additional packages mainly *spacetime* [24], *gstat* [25] and *plotKML* [26] with the usage of IDE RStudio. The final KML files were displayed and analysed in the free version of Google Earth™. The overall schema of the processing workflow that is visually described step by step is depicted in Figure 1.

## Methods and materials

### Google Earth™ and Keyhole Markup Language

Google Earth™ is freely available (although proprietary) 3D virtual globe provided by Google Inc. that allows browsing the geographical data in exchange formats. The technology fuses imagery, terrain, and GIS data to deliver them to their users by means of a client–server architecture, where a Web browser is the client that accesses the data viewing and navigational services on the Google Earth™ server [5]. It enables the interactive displaying and exploring of spatial and spatio-temporal data including the zooming, querying, adding overlays or animations. However, the strength of Google Earth™ is not the data creation, but their visualisation. The free version of Google Earth™ has a limited number of data file formats that can be opened, including images formats, GPS formats, COLLADA models and mainly Keyhole Markup Language files (KML/KMZ).

Keyhole Markup Language (KML) is a file format used to display geographic data in an Earth browser such as Google Earth™ or Google Maps. KML uses a tag-based structure with nested elements and attributes and is based on the XML standard [27]. Moreover, KML is also the exchange standard for geospatial data approved by Open Geospatial Consortium. The KML file specifies a set of standard features (e.g. geolocation, placemarks, images, polygons, 3D models, textual descriptions, timestamps) for the display in Google Earth™ [28].

Main reasons, why to use the combination of Google Earth TM and KML, are well described in [26, 29] and may be summarized as accessibility and popularity of Google Earth™; availability of good-quality (geo)data as base layers; KML as OGC standard for the geodata; and variability of KML that provides cover platform for various data types and their visualisation.

### Surveillance and spatial data

**Figure 2:**
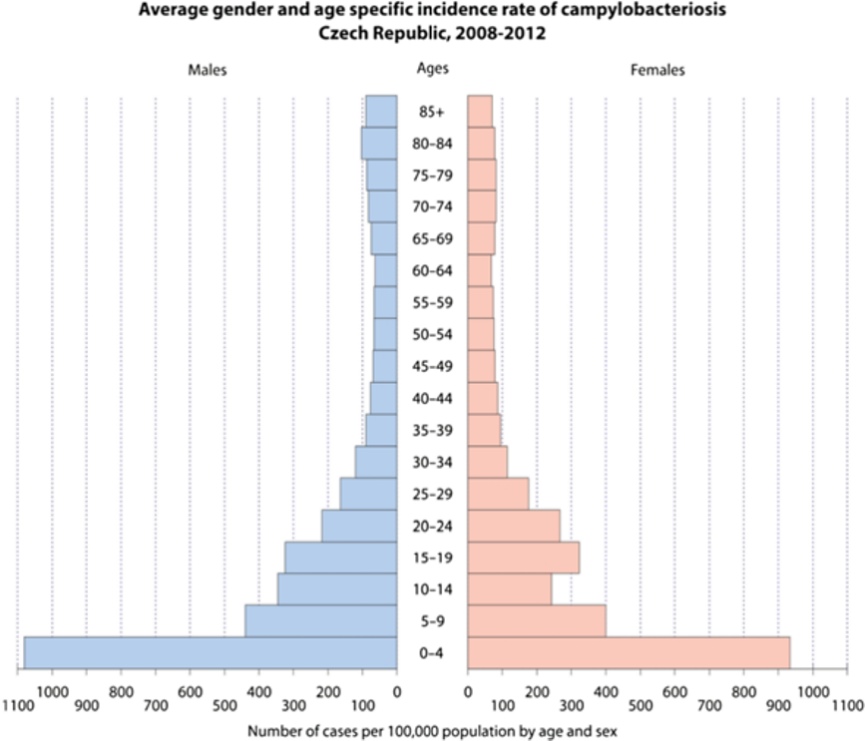
**Average incidence of campylobacteriosis in the Czech Republic (2008–2012) by age and gender.**

The dataset used in this study was provided by The National Institute of Public Health of the Czech Republic. The data come from the EPIDAT database, which is the official database ensuring the mandatory reporting, recording and analysis of infectious diseases in the Czech Republic. The database contains almost 100,000 cases of Campylobacteriosis infection in the Czech Republic between 1 January 2008 and 31 December 2012. The database is filled directly by physicians. The dataset does not contain any confidential information (name, identity number, full address) that would allow the re-identification of the individual. In order to geocode data to the street level, we used the geocoding function implemented in the *R language* script [30] using the Application Programming Interface (API) of the Czech web maps provider Mapy.cz. This API does not have any day limits, but it is usable mainly in the area of the Czech Republic. Surveillance data were categorized according to the age/sex structure provided by census data and demography data supplied by Czech Statistical Office. Figure 2 shows the stratified average year incidence in the Czech population based on the data from 2008–2012. Children under four years of age are the most affected demographic group, but increased incidence appears in the group of children and youth younger than 20 years old. People in age groups older than 30 years are the least affected. The incidence rates in these age groups do not exceed 100 cases per 100,000 people. The average year incidence of the Campylobacteriosis in the Czech Republic in 2011 was 225 cases per 100,000 population [31]. Up to 72% of municipalities were affected by the disease in 2008–2012 with the incidence rate ranging from 0 up to 7,892 cases per 100,000 population, with up to 480 cases recorded within one year in individual municipality. Table 1 provides further statistical characteristics. Additional file 1 shows annual changes in the incidence rate in municipalities in the animated map.

Data were spatio-temporally aggregated (weekly data in regular grid/municipality), in order to enter spatio-temporal kriging and space-time scan statistics. This step also reduced the influence of administrative borders and provided the possibility to present results in a finer resolution. We chose the square grid covering the Czech Republic with the 4 km^2^ cell size. On one hand, it provides suitable spatial resolution but preserve the data confidentiality on the other, while it is still computationally effective. Moreover, previous studies showed that the spatial autocorrelation between individual points of infectious disease is usually strongest in distances around 2 km [32]. Final aggregated data consist of 261 time cuts representing weeks and 6,385 administrative units/34,440 grid cells.

### Bubble chart in Google Earth™ as an alternative to space-time cube

The confident nature of the data does not allow the visualisation of disease cases in the form of precise dot maps due to the information confidentiality. That is why the aggregation of the data is necessary. We aggregated frequency of disease cases in both, space (the regular grid) and time (weekly cases). This kind of aggregation enables the displaying of data as circles in map or spheres in 3D environment. The size and colour of the sphere correspond to the frequency of disease occurrence in individual grid cell. The time domain occurs in two forms in this kind of visualisation. Firstly, there is an internal time component describing the precise data and allowing the time animation. Secondly, the time supplies the z-axis of the case frequency in the grid cell; i.e. offset from the surface. By this manner, we are able to visually explore time trends of disease behaviour in individual localities, as well as to compare group of localities in space (in particular time slices) and in time (3D view on selected zoom level). The presented technique can be considered to be a variation on the well-known space-time cube model [33, 34]. Time support and the length of the time period can be easily set using the incorporated time slider that also enables the animation of the phenomena.

### Spatio-temporal kriging: the joint power of space and time

While the kriging is a well-known and well-described interpolation method [29, 35] that has been used in geosciences for several decades, its spatio-temporal enhancement is rather a new procedure. The idea of spatio-temporal kriging regularly appears for several decades, but its computational demands allowed the proper implementation of the method only recently thanks to the increasing computing performance of information technologies. The spatio-temporal kriging uses correlation of the data evaluates by the spatio-temporal variogram that describes spatial, temporal and also joint spatio-temporal correlations of the data [36]. Due to its novelty, the method is used very rarely in the context of health data, e.g. in [37, 38].

**Figure 3:**
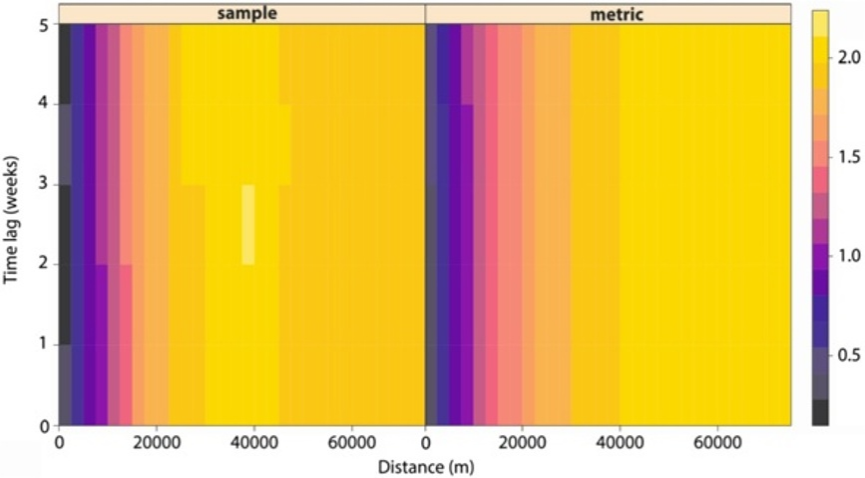
**Empirical spatio-temporal variogram and fitted theoretical spatio-temporal variogram. Empirical spatio-temporal variogram (left part of the image) describes spatial, temporal and also spatio-temporal relations that can be found in the sample data.** The fitted theoretical spatio-temporal variogram (right part of the figure) shows the fitting of the theoretical metric model that tries to describe all relations by mathematically defined function with estimated parameters. The horizontal axis shows the distance among data points in space; vertical axis displays the time distance and semivariance (the power of the relations) is expressed by the colour scale. The theoretical model approximates the real data mainly at closer distances in both, space and time.

The main aim of the spatio-temporal kriging in this study was to create the continuous surface of the disease incidence in the populated places of the Czech Republic in every time unit given by the data aggregation. The logarithm of standardized incidence serves as input data, and the metric model of spatio-temporal variogram was used in the computation. To be more particular, we used exponential model with following parameters: *nugget = 0.15, partial sill = 1.94, range = 14150.46 m and space-time anisotropy = 544.58*. Figure 3 shows the visualisation of the empirical spatio-temporal variogram that directly depicts spatial dependence in both, space and time using the colour scale. It also depicts the fitted theoretical model of the spatio-temporal variogram. The theoretical model is well fitted mainly in the left part of variograms, which means that the best estimations are made for observations closer in space and time. The interpolated continuous incidence surface was computed by ordinary global spatio-temporal kriging on point support coming from the centroids of the aggregated data.

### Space-time clustering

The spatio-temporal scan statistics, that had the aim to identify clusters of high and low rate areas together in the continuous geographical regions and time, was computed in the environment of the SaTScan 9.3 software [39]. This procedure served to confirm that patterns in the data are significant real world situations, and they are not just the realization of a random process in the study area. Input data consist of age/sex stratified individual cases aggregated in municipalities by weeks; municipality demography structure and coordinates of centroids of administrative units. The space-time retrospective analysis of high and low rate clusters was based on the age/sex stratified data with Poisson probability model. The SaTScan was set to find clusters of maximum size of 3% of the population in the circular window [40] with maximum temporal cluster size set to 50% of the time period or 100% in case of purely spatial clusters. The nonparametric temporal trend adjustment with time stratified randomization [41] was also applied to ensure the comparability of rates within various periods. The significance of found clusters was assessed at p-value lower than 0.05 and performed by 999 Monte Carlo realizations. Then, the program calculated indirectly standardized rates (expressed as the relative risk which is the observed rate divided by the expected rate) for each identified geographic cluster [42] and only significant clusters remained in the outputting files.

## Results

### Geovisualisation of the surveillance frequency data

**Figure 4:**
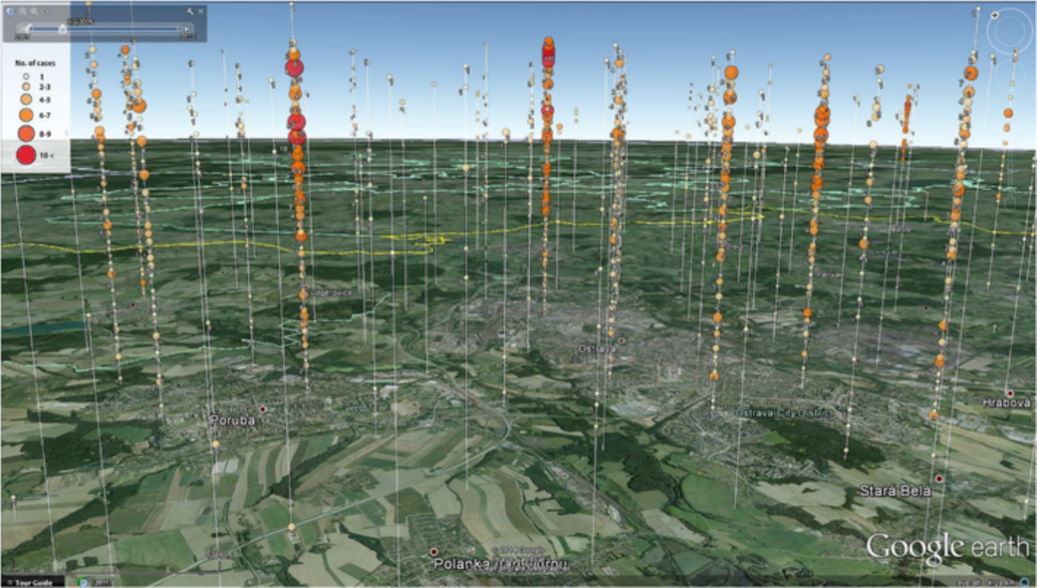
**Spatio-temporal bubble chart visualised in Google Earth.** Number of cases per week are visualised using the bubble chart in the environment of Google Earth. The data are aggregated in the regular square grid (4 km^2^). The number of cases is represented by the size and colour of the sphere as well as by the neighbouring number. The time serves as an offset from the terrain. The time slider is located in top left corner. It enables the settings of the date and also the period of visualised data. Visualised area belongs to the north-eastern part of the Czech Republic near Ostrava city that is one of the highly affected areas. See ’Additional file 2’ for a short example.

The first visual overview of the space-time pattern in the data was realized using the KML file that contains the information about weekly frequency of the disease occurrence within the regular grid. The information is visualised as 3D spatio-temporal bubble chart (Figure 4). The size and colour of bubbles depict the frequency of cases in the grid cell in individual weeks in order to distinguish between the actual disease’s occurrence in selected time intervals and areas easily. The elevation above the surface is then linked to an individual week, i.e. there are 261 levels, where bubbles can or cannot appear during weeks. There are labels next to each bubble and the guideline in order to ensure the proper reading of the number of cases represented by the size of the bubble, as well as the membership to the appropriate grid cell. The red colour depicts the category with the highest frequency in order to attract the user’s focus immediately. The time slider located in the top left corner of the working environment enables both, the setting of the time period and the length of its lasting. Using this feature, one can geovisually analyse the overall area and also the specific location. In fact, there is a possibility of the evaluation of the distribution in individual time slices, locations or their combinations. Additional file 2 shows how the created KML file looks and how it is possible to work with it.The example of the visualisation is depicted on Figure 4 that shows one of the areas with the highest occurrence of the campylobacteriosis. Using the visual analytics, we identified several areas with higher frequency of the disease’s occurrence. The eastern part of the Czech Republic (Moravia) is more affected than the western part (Bohemia). Particularly, Campylobacteriosis appears mainly the north-eastern part of Moravia and then southern part of Moravia. Moreover, three small clusters of increased occurrence were visually identified near Bohemian cities Prague, Pilsen and Ceske Budejovice. The central part of the study area seems to indicate rather a sparse occurrence of the disease.

### Geovisual analytics of the continuous incidence surface

**Figure 5:**
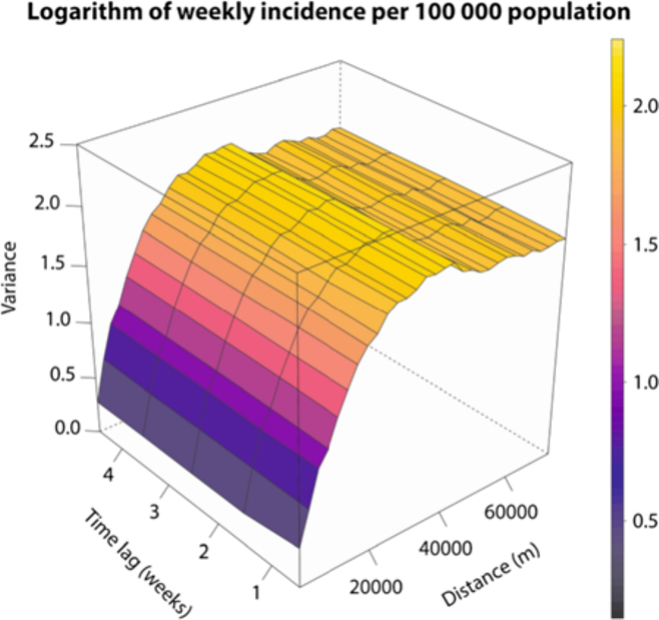
**Spatio-temporal variogram used for the interpolation.**

**Figure 6:**
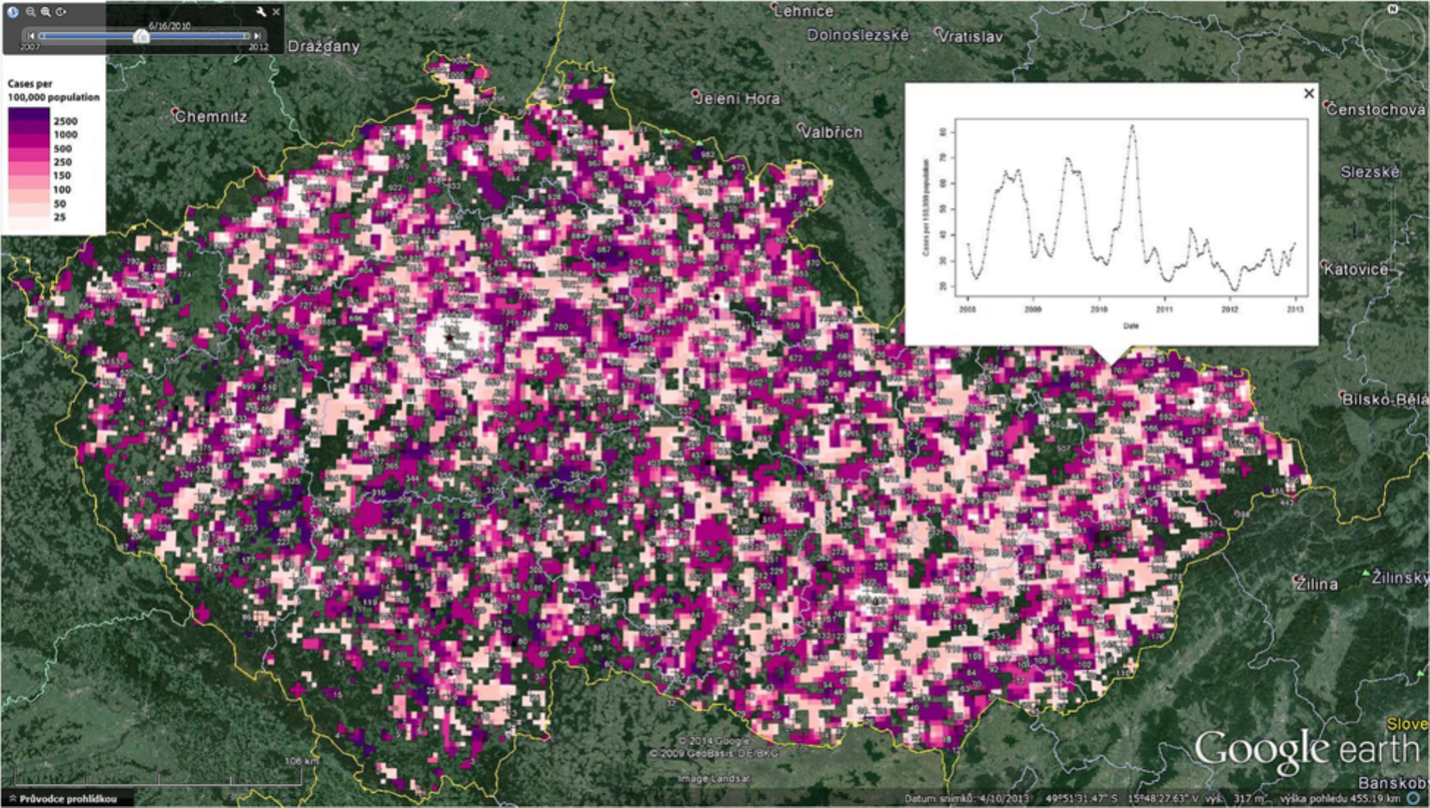
**Continuous spatio-temporal surface of the incidence in the Czech Republic.** The spatiotemporally interpolated surface of the incidence in the Czech Republic is classified in nine categories that are represented by the colour scale that is also located in the figure. One can also easily explore different time periods using the time slider. The surface is depicting only places that are inhabited. The visualisation contains also 1,000 sample points that allow to display the time-series graph of the incidence rate on the location. See ’Additional file 3’ for a short example of the animation using KML and Google Earth.

Because we used the spatio-temporal kriging as the method of interpolation, the spatio-temporal variogram was created (Figure 5) prior the interpolation proceeded. This variogram described the spatial, temporal and spatio-temporal dependencies. It was found out that spatial dependencies among incidence rates are the strongest, and likely the most meaningful within 14 km range in space and within four weeks interval in time. These settings were used for the consequent interpolation. These data were categorized and exported into KML file in order to enable visualisation in the Google Earth™ software. It allows interactive exploring of the spatial and temporal support of the data including the settings of the scale and time interval or the animation. The file consists of 261 raster layers representing each week during the study period. Furthermore, the visualisation of continuous incidence surface in KML is enriched by thousand random sample points that carry the time series plot of the incidence in selected location (Figure 6). The results of the interpolation are classified into 9 categories (<25; 25–50; 51–100; 101–150; 151–250; 251–500; 501–1,000; 1,001–2,500; >2,500 cases per 100,000 population) according to the incidence rate in the cell. The legend remains the same for every time interval, so the state of the phenomena can be easily compared in time (using the time slider in Google Earth™) and space. The KML file also contains time-series graphs of the incidence rate for sampled locations that allows better evaluation of the disease occurrence. Thus, the user is able to identify both, expected patterns and unexpected findings and compare them immediately with situation in different locations and their neighbourhood. Additional file 3 shows how the created KML file looks and how it is possible to work with it.

The visual analytics helped to identify several findings. Some of them came directly from the methodology and generally accepted knowledge about the Campylobacteriosis, e.g. seasonality of the disease with the peak during summer months (June–August). The change of the incidence caused by the seasonality is usually less evident in the densely populated areas. On the contrary, it is more apparent in rural areas and also in bigger towns’ neighbourhoods that are often used as recreational areas. The increased incidence rates are also visible in mountain areas during the winter season, which is valid mainly for the foothills of the two biggest mountain ranges – Krkonose Mountains and Jeseniky Mountains.

### Geovisualisation of space-time clusters

**Figure 7:**
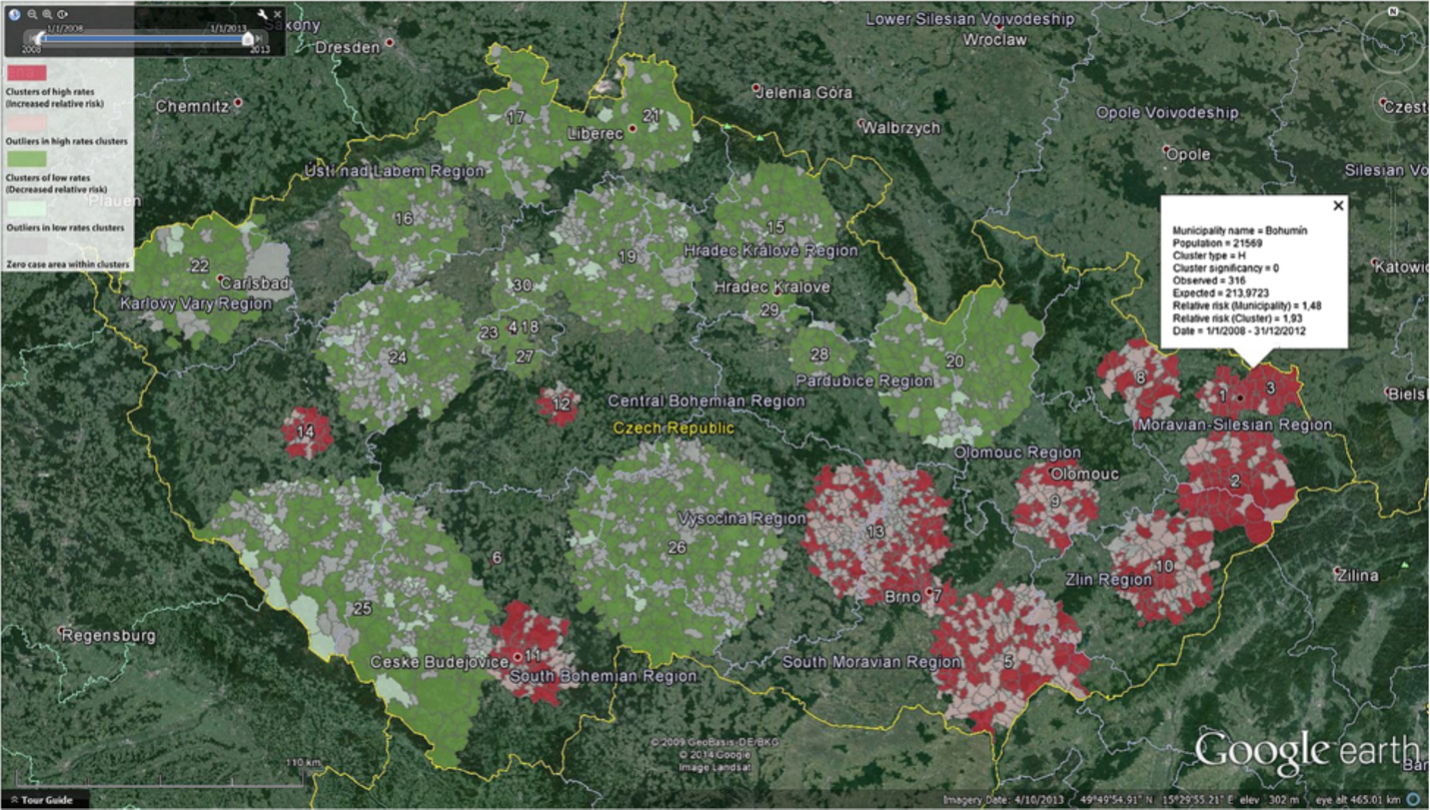
**High and low rates space-time clusters of campylobacteriosis in the Czech Republic.** Dark red colour depicts clusters of more affected/vulnerable municipalities. Clusters that are more resistant to the campylobacteriosis than its neighbourhood are dark green. The map also shows the particular heterogeneity of clusters. The healthy areas within more affected clusters are coloured in light red, and on the contrary, more affected municipalities in healthy clusters are depicted as light green. The overall description of individual clusters is in Table 2.

The spatio-temporal scan statistics using SaTScan software is also able to generate results as KML files. They are usually made up of the indexed circles representing detected clusters according to their type, and they also contain the centroid of municipality units. We used this primary information in combination with the original municipal data. Then we generated resulting KML, which consists of municipalities coloured by the membership to low/high rates clusters or to outliers. During the evaluation of clusters, one should focus not only on characteristics of individual clusters but also on their inner homogeneity. The map in Figure 7 depicts the location and type of clusters and also their structure. Outliers that cause the heterogeneity are visualised in lighter colours while areas without any disease occurrence are depicted in grey. Outliers in the high rates clusters (light red coloured areas) are municipalities that have an average or low relative risk (RR ≤ 1.50) although they belong to the high rates cluster. On the contrary, outliers in low rates clusters (light green coloured areas) are municipalities that have average or high relative risk (RR > 0.80) although they belong to the low rates cluster. Uncoloured areas on the map then represent municipality that does not belong to any cluster. The final KML is also enriched by the characteristics of individual municipalities and by the time stamp that allows usage of the time slider and animation like in the previous examples.

During the study period, we identified up to 30 significant clusters (p-value < 0.001) in the Czech Republic. Fourteen of them are clusters of high rates that signalize areas with increased risk estimates (RR > 1.50). The primary most likely cluster is the cluster number one (RR = 2.16) that lies in the north-eastern part of the Czech Republic in the city of Ostrava (Figure 6). It consists of thirty-one municipal districts, which cover almost 293,000 of the population in the risk. Other clusters are so-called secondary clusters. Nine of all high rates clusters are located in the eastern part of the Czech Republic called Moravia. Only five high rates clusters are located in the Bohemia (western part of the Czech Republic). Most of the high rates clusters show throughout the entire study period, while only five of them (no. 5, 10, 11, 13, 14 in Table 2) are more specific showing the particular outbreak or period with an increased risk of the campylobacteriosis. There are also two secondary clusters of high rates that cover only one administrative unit. First of them is the very centre of Prague (RR = 4.13), i.e. densely populated area, second is a small village in the South Bohemia called Drazic (RR = 41.92). The rest of detected clusters (n = 16, RR ≤ 1.80) are low rates clusters, i.e. they represent the area where the risk estimation is lower than expected. All low rates areas are located in the Bohemia; the only exception is cluster no. 20 that also covers part of Moravia. One can find two main types of low rates clusters – the first type can be described as mainly mountainous areas with low population density (no. 15, 17, 20, 21, 25, 26); the second type then consists of densely populated areas with lower agricultural activity. The description of all identified clusters is stored in Table 2 including the cluster type, period of cluster duration, number of municipalities within the cluster, observed and expected cases, relative risk and most potentially affected population.

**Table 2:** **Space-time clusters of high and low rates of campylobacteriosis in the Czech Republic, 2008–2012**

Cluster	T ^1^	Time ^2^	Region ^3^	C ^4^	Ob ^5^	Exp ^6^	RR ^7^	Population ^8^
1^*^	H	2008/01/01 – 2012/12/31	Ostrava	31	5975	2861	2.16	292,978
2	H	2008/01/01 – 2012/12/31	North Wallachia - Lachia	70	5414	2788	2.00	277,236
3	H	2008/01/01 – 2012/12/31	Havirov and Karvina	16	4773	2534	1.93	256,657
4	H	2008/01/01 – 2012/12/31	Prague - centre	1	1006	245	4.13	29,948
5	H	2008/05/13 – 2010/11/01	Southern Moravia	167	2274	1432	1.60	292,885
6	H	2008/01/01 – 2012/12/31	Drazic	1	72	2	41.92	214
7	H	2008/01/01 – 2012/12/31	Brno - city	19	3951	2590	1.55	271,742
8	H	2008/01/01 – 2012/12/31	Opava	37	1714	877	1.97	87,203
9	H	2008/01/01 – 2012/12/31	Hanakia	66	3828	2526	1.54	256,721
10	H	2009/04/14 – 2011/09/05	Southern Wallachia	90	1596	932	1.72	196,522
11	H	2010/01/12 – 2010/02/22	Ceske Budejovice	60	194	36	5.41	157,425
12	H	2008/01/01 – 2012/12/31	Benesov	15	640	313	2.05	31,115
13	H	2010/04/06 – 2010/10/04	Brno - surroundings	224	568	286	1.99	284,346
14	H	2011/05/03 – 2011/11/14	Pilsen	22	394	201	1.96	197,263
15	L	2008/01/01 – 2012/12/31	Krkonose mountains	128	997	1841	0.54	182,641
16	L	2008/01/01 – 2012/12/31	North-Western Bohemia	108	1853	2930	0.63	290,222
17	L	2008/01/01 – 2012/12/31	Usti nad Labem - Decin	93	1266	2958	0.42	288,203
18	L	2008/01/01 – 2012/12/31	Prague - East	4	1591	2530	0.62	280,780
19	L	2008/01/01 – 2012/12/31	Mlada Boleslav	173	1124	2590	0.43	256,738
20	L	2008/01/01 – 2012/12/31	East Bohemia/Moravia borders	138	1482	2571	0.57	253,941
21	L	2008/01/01 – 2012/12/31	Jizera Mountains	59	1302	2320	0.56	230,360
22	L	2008/01/01 – 2012/12/31	Carlsbad	82	1284	1992	0.64	202,256
23	L	2008/01/01 – 2012/12/31	Prague - West	16	1805	2961	0.60	305,103
24	L	2008/01/01 – 2012/12/31	Kladno – Beroun - Rakovník	172	1950	2684	0.72	268,391
25	L	2008/01/01 – 2012/12/31	Bohemian Forest	211	1578	2667	0.58	268,701
26	L	2010/11/23 – 2011/04/25	Vysocina	252	84	247	0.34	294,203
27	L	2008/01/01 – 2012/12/31	Prague – South-East	21	1821	2961	0.61	318,958
28	L	2008/01/01 – 2012/12/31	Vysoke Myto	31	271	509	0.53	49,304
29	L	2010/11/09 – 2012/06/11	Hradec Kralove	25	173	348	0.50	113,501
30	L	2008/01/01 – 2012/12/31	Neratovice	71	2000	2490	0.80	249,994

*Denotes primary cluster; p-value of all clusters is < 0.001; ^1^the type of the cluster: H stands for high rates clusters (high relative risk) and L stands for low rates clusters (lower relative risk); ^2^Time describes the period of cluster’s duration; ^3^Regions named by the local names of town, area or mountain range; ^4^the count of municipalities in the cluster; ^5^the observed number of cases in the cluster; ^6^the expected number of cases in the cluster; ^7^computed relative risk; ^8^estimated population in the cluster.

## Discussion

### Strengths and limitations of Google Earth™ and KML in the field of geovisual analytics

The case study provided three main results, (1) the spatio-temporal bubble chart; (2) the spatiotemporally interpolated incidence surface; and (3) detected spatio-temporal clusters of high and low rates. KML files were created from all results, and then they were visualised in Google Earth™ with the purpose of following geovisual analytics. We are aware that Google Earth™ is not the complex platform for the overall process of geovisual analytics covering all necessary steps from data uploading, their transformations, analyses, up to final presentation and dissemination. Reasons for this are mainly due to different tools that pre-process data and create output files and also because of the limited data analysis capability of Google Earth™. On the contrary, the advantages of the Google Earth™ are undisputable. Google Earth™ is multiplatform, freely available and extremely wide-spread (more than 1 billion downloads in the year 2011 [19]) application, which makes it probably the world’s most used browser of geodata. The Google Earth™ interface is also user-friendly and intuitive, so users do not need any specific knowledge. The visualisations in Google Earth™ are usually interactive using the zooming and simple querying functions on displayed objects. The crucial aspect of Google Earth™ concerning the geovisual analytics is the direct support of spatio-temporal data and their animations. This aspect helps to fulfil one of the main ideas of the geovisual analytics: *“Detect the expected and discover the unexpected”* [1, 43]. It opens the geovisual analytics not only for specialized researchers, but also to decision-makers or to the general public, which makes the dissemination of results much easier. However, the geovisual analytics in Google Earth™ often requires a certain level of user’s experiences. The other advantage is the usage of KML as the primary format of input data. The KML is an open standard for geodata and provides the broad range of possibilities for the visualisation. KML can contain different kinds of data formats, or it can link to them. It might cause an increased computer’s memory usage mainly in the case of big datasets consisting of vector data or a series of raster maps. However, KML files can be compressed to KMZ, which is the zipped version of KML that provides reasonable savings of the hard-disk space. In case that someone needs the linked view consisting of several types of information, it is possible to create such kind of presentation using KML. It is right that proceeding of all analyses requires several prerequisite and data preparation. The subsequent creation of resulting KML files is, in fact, quite simple. In the presented study, KML files were made and customized mainly using R package plotKML, which is very straightforward and not difficult to use (considering elementary skills in R language). However, KML can be created directly from spatial data using geoinformation system (e.g. QGIS, ArcGIS for Desktop) very easily. SaTScan also supports the creation of KML files showing identified clusters as one of its results.

### Spatio-temporal bias in the data

We mentioned that our case study had purely spatial, temporal and spatio-temporal character, so the underlying environmental and social factors were not included. However, we are aware of the fact that the number of factors may be significant for the distribution of diseases. Relations of these factors on the spatial distribution of campylobacteriosis that we analysed is well-described in previous studies [42, 44–46]. Together with the geographical knowledge of the study area, the visual analytics of the disease incidence surface and detected clusters can point out the likely connection among the areas with increased risk and agriculture activities, rural areas, social deprivation and demographic structure of the population. Researchers should be also aware of the spatial and temporal variability of particular diseases and their clusters that may be closely related to changes in environmental and demographic factors (climate change, population change, land use change, etc.).

Since the presented case study and its results are focused mainly on the spatial and spatio-temporal properties of the disease distribution, the selected spatial, and temporal scale are very important parts of all procedures, whether they are dealing with the aggregation, range of clusters, estimation of parameters during spatio-temporal kriging or with resulting visualisations and their understanding. The scale of the analysis or the level of aggregation is usually a trade-off between specificity and precision: the smaller the area, the more accurate and relevant are the findings to the local population, but the greater are the imprecision and the potential for bias [47]. Furthermore, many datasets exhibit different spatial patterns when viewed at one spatial level compared to another, which is known as a ’scale’ effect [48]. The temporal scale of the aggregated data was constantly set to weeks throughout the study. However, we used two different spatial types of the aggregation - the municipality level and the regular grid. The main advantage of the analysis in the municipality districts is the known demographical structure of the population, which means more accurate rate estimates. On the contrary, the population structure of a regular grid is only estimated, so the rates carry more uncertainty. However, this method creates smoothened surfaces that decrease differences appearing among neighbouring administrative units, and it also provides more detailed results.

### Why (not) to use spatio-temporal kriging and scan statistics?

The continuous incidence surface represents the estimate of the incidence rate of campylobacteriosis in populated places in the Czech Republic during every week of the study period. On one hand, it expresses the incidence rate also in places without any recorded case of the disease. Contrarily, the interpolation can suitably describe the state of the situation and the progress of the disease distribution simultaneously in space and time. However, it is always necessary to count with the certain amount of inaccuracy of results due to the expert estimation of interpolation parameters. The incidence surface confirmed several well-known facts; e.g. more stable estimates are gained in densely populated areas; peaks of the disease occurrence usually appear during summer months and others. It also helped to identify locations with opposite trend or locations with more than one peak. It is necessary to notice that the computation of both, spatio-temporal variogram and kriging interpolation, are very computationally demanding. The computation of spatio-temporal variogram took 35.4 hours (Intel Core i7-3770 CPU 3.90 GHz, 8 GB RAM). Firstly, the calculation of the kriging was not possible to proceed to the entire area of the country, but the usage of looping functions with sets of reduced areas allowed the interpolation, which lasted 13.7 hours. The output raster dataset was then clipped by the layer of populated areas in the Czech Republic, which was based on the CORINE land cover dataset [49].

The spatio-temporal scan statistics [39], which is commonly used for spatio-temporal cluster analysis, has several advantages: it conforms to the population density and confounding variables such as age and sex, and there is no pre-selection bias because groups are searched without prior assumptions about their location period, size or time [50]. This statistical method takes into account multiple testing; allowing us to obtain a single p-value, and it locates and specifies the occurrence of the clusters. Unfortunately, the influence of the parameters settings in SaTScan is explored only partially so the maximum spatial cluster size, time window as well as adjustments were selected experimentally but with regard to findings of previous studies [40, 51]. We also tested the alternative scan statistics settings of scan statistics in order to compare the validity of results. Various combinations of population in risk (3%, 5%, 10% and 50%), maximum cluster size (30 days, 105 days and 50% of time period) and temporal trend adjustments but the results did not differ significantly. Logically, the number of clusters was different – the higher population in risk, the lower number of larger clusters. However, the locations of main clusters were very similar as well as the period of their appearance.

## Conclusions

The analysis of spatio-temporal data often happens conditionally, meaning that either first the spatial aspect is analysed, after which the temporal aspects are analysed, or vice versa, but not in a joint, integral modelling approach, where space and time are not separated [52]. The presented study combines results of truly spatio-temporal methods evaluates mutual interactions in both dimensions (space and time) and their visualisation in Google Earth™ that provides the suitable environment for geovisual analytics. By means of usage Google Earth™ as visualisation medium for results, we gained the additional value to all analyses performed. The results incorporate not only spatial component as it is common, but also the time dimension, both at once. Hence, it is desirable to explore them in fully-fledged environment as Google Earth™ that allows seamless browsing through space and time. Using the KML files as the basis for geovisual analysis, analyst can provide results and their possible interpretations in an attractive and self-explaining form that is accessible not only to specialized researchers, but also to wider audience without any additional specific knowledge. Google Earth™ is presented in the study as a tool that allows perceiving the expected and discovering the unexpected patterns in space and time. To be more specific, we provided (1) visualisation of surveillance data in three-dimensional bubble chart map; (2) visualisation of spatio-temporal interpolation of incidence rate in the form of time slices suitable for animations; and (3) visualisation of identified spatio-temporal clusters. We could have explored time trends of disease behaviour in individual localities visually. We also could have compared a group of localities in space (in different time slices) and in time (using 3D view on selected zoom level in a certain locality). All analyses and their results visualised in Google Earth™ proved themselves as efficient tools for the exploration of the spatio-temporal patterns of disease distribution, which may help researchers to identify sources, outbreaks and progress of particular diseases. We can suggest Google Earth™ as the platform that is usable for the geovisual analytics, nevertheless it is still needed to combine it with pre-processing tools that prepare the data into a form suitable for the geovisual analytics itself.

The results of the geovisual analytics identified periodical patterns in the behaviour of the disease with an increased incidence during summer months in both, hinterland areas of regional centres and areas used for the recreation. On the other hand, it also identified secondary peaks of the incidence during the winter in the foothills of mountains. The spatio-temporal scan statistics recognized fourteen clusters of municipalities with increased vulnerability (RR ≥ 1.50) to the campylobacteriosis and sixteen clusters of healthier municipalities (RR ≤ 0.80). Detected clusters divided the Czech Republic into two dissimilar geographical units – more affected Moravia (eastern part of the Czech Republic) and less affected Bohemia (western part).

Future steps of the work will involve the modelling of the disease distribution using socio-economic and environmental factors focusing mainly on areas identified as high rates clusters. We also want to incorporate of the subsequent visualisation of modelling results in the geovisual analytics procedure.

## Electronic supplementary material

Additional file 1: **Animated map of annual changes in the incidence rate in municipalities of the Czech Republic, 2008–2012.** (GIF 6 MB)

Additional file 2: **Example of the spatio-temporal bubble chart visualised in Google Earth using KML.** (MP4 13 MB)

Additional file 3: **Example of the continuous spatio-temporal surface of the weekly incidence in the Czech Republic, 2008–2012.** (MP4 13 MB)
