# Supplementary figures and images for: Using geovisual analytics in Google Earth to understand disease distribution: a case study of campylobacteriosis in the Czech Republic (2008–2012)
Source: Int J Health Geogr. 2015 Jan 28;14:7. doi: 10.1186/1476-072X-14-7 (PMC4328415; doi:10.1186/1476-072X-14-7)

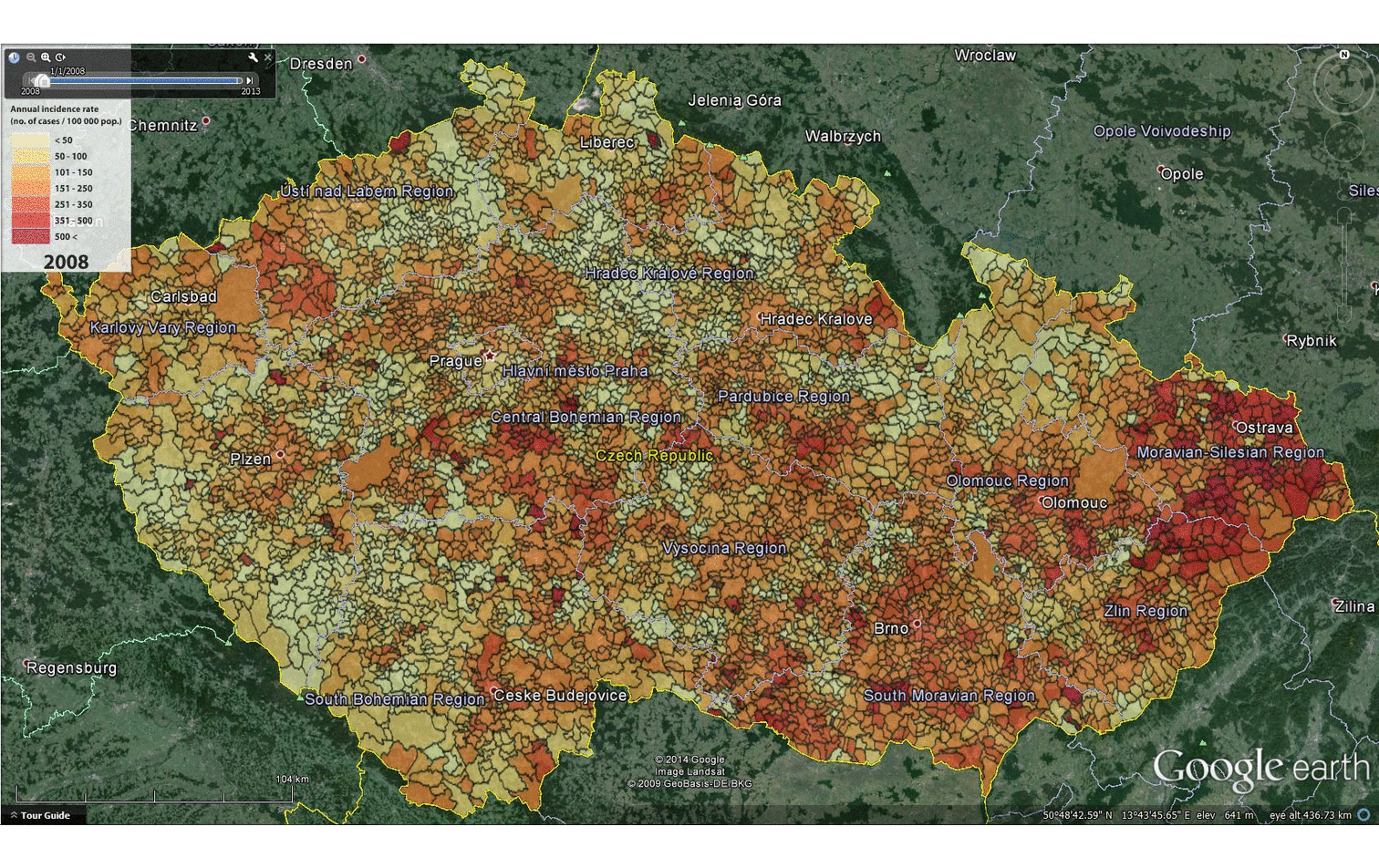

Supplement: Supplementary file 1 — Additional file 1: Animated map of annual changes in the incidence rate in municipalities of the Czech Republic, 2008–2012. (GIF 6 MB) [file 12942_2014_626_MOESM1_ESM.gif]
